# Decomposing the urban–rural inequalities in the utilisation of maternal health care services: evidence from 27 selected countries in Sub-Saharan Africa

**DOI:** 10.1186/s12978-021-01268-8

**Published:** 2021-10-30

**Authors:** Oduse Samuel, Temesgen Zewotir, Delia North

**Affiliations:** School of Mathematics, Statistics and Computer Science, University of KwaZulu-Natal, Durban, 4001 South Africa

**Keywords:** Antenatal care, Delivery place, Fairlie decomposition, Healthcare Health professionals, Pregnancy, Sustainable development goal

## Abstract

**Background:**

There has been a substantial improvement in reducing maternal mortality in the Sub-Saharan African region. The vast rural-urban gap in maternal health outcomes, however, is obscured by this average achievement. This study attempts to measure the contribution of identified risk factors to describe the average rural-urban difference in the use of antenatal care, health facilities for delivery, and health professional assistance at delivery.

**Method:**

To achieve this objective, we used descriptive analysis and Fairlie non-linear decomposition method to quantify covariates’ contribution in explaining the urban–rural difference in maternal healthcare services utilisation.

**Result:**

The study’s finding shows much difference between urban and rural areas in the use of maternal healthcare services. Socio-economic factors such as household wealth index, exposure to media, and educational level of women and their husbands/partners contributed the most in explaining the gap between urban and rural areas in healthcare services utilisation.

**Conclusions:**

Interventions to bridge the gap between urban and rural areas in maternal healthcare services utilisation in Sub-Saharan Africa should be centred towards socio-economic empowerment. Government can enforce targeted awareness campaigns to encourage women in rural communities in Sub-Sharan Africa to take the opportunity and use the available maternal health care services to be at par with their counterparts in urban areas.

## Plain Language summary

Maternal health refers to the health of women throughout pregnancy, delivery, and the postnatal period. Each step should be a good experience that ensures mothers, and their infants realize their maximum health and well-being potential. In this study, we used individual, demographic, and socio-economic characteristics to measure the urban–rural discrepancies in maternal health care services in Sub-Saharan Africa. We used Information of 220 164 women of child-bearing age (15–49) gathered from National Demographic Health Surveys from 27 countries in the Sub-Sahara African region. We found 46.1% of women in rural areas had no education, 39.7% of the women in rural areas have husbands/partners with no education, and 60.1% of the women in rural areas are from households with poor wealth indexes. The use of maternal health care services found to be predominant in the urban areas than rural areas, and the measure of this difference can inform policymakers on the level of effort that needed to be put in place to balance the discrepancies and improve maternal health in general.

## Introduction

Maternal and child health care are among the Sustainable development Goals [1]. Countries are making efforts to achieve this target by creating policies to reduce deaths due to pregnancy complications and childbirth complications. According to the United Nations inter-agency figures, the global maternal mortality ratio decreased by 38% from 342 deaths to 211 deaths per 100,000 live births between 2000 and 2017 [2]. In 2017 alone, Sub-Saharan Africa accounted for about 66% (254,000) of the estimated global maternal deaths (196,000) [3]. The increased rates of maternal and neonatal mortality in the Sub-Saharan African region are primarily due to the inadequate health care facilities and accessibility of the health care service or even the lack of utilisation of available health facilities for delivery by pregnant women for reasons such as religious beliefs, cultural customs or lack of funds to access the health care services [4].

Maternal health care utilisation remains the priority in decreasing the risk of diseases, haemorrhage, and death from pregnancy and childbirth complications [5]. Antenatal and delivery care are both vital to maternal and infant wellbeing. Appropriate maternal health care can undoubtedly avoid adverse maternal and new born pregnancy outcomes, mostly by preventive intervention or successful management [6]. Health professionals commonly advise that antenatal visits commence early in pregnancy and proceed at frequent intervals during pregnancy to reduce possible pregnancy risks [7]. Also, mothers need to deliver their babies in hygienic conditions with adequate equipment and materials and in the presence of a trained health worker to minimise the risk of infections and ensure that these professionals adequately handle any complications that may arise. The postnatal period which is the period shortly after giving birth is also a risky period for both mother and child. The period has a higher risk of mortality; therefore, adequate postnatal care is critical in reducing mortality and improving quality of life [8].

Despite overwhelming evidence from the literature on the positive effect of maternal health care services in mitigating the risk of deaths due to pregnancy and childbirth complications, most developing countries continue to record low access to these services [9–11]. Facility-based delivery has been proven to be an essential factor in preventing maternal death. Yet, a significant number of women in Sub-Saharan Africa still deliver at home without health professionals’ assistance [7, 12, 13].

In both industrialized and developing nations, a large body of literature has documented the disproportionate usage of maternal health care services among women of various socioeconomic levels, and rural-urban residences [14–17]. Women from higher socioeconomic classes are more likely to utilize healthcare facilities, and over the years, the economic inequality in the use of healthcare services has widen [18–20]. Despite overwhelming evidence of geographical and economic disparities in maternal health, not much Information is available on the urban–rural differences in maternal health care services. Furthermore, the impact of various factors in determining the rural-urban disparity in maternal health care services usage has not yet been fully investigated.

Several factors contribute to women’s better health in urban settings. Women in urban regions have easier access to health facilities and maternal health intervention programs [21–23]. The makeup of the urban population in terms of wealth index, educational status, and other socioeconomic characteristics may also have a beneficial impact on the use of maternal health care services [24–26]. Research in Sub-Saharan Africa has revealed that women in urban regions utilize maternal health care more. Still, the literature on urban–rural disparities in the usage and the role of various factors in determining these discrepancies is sparse.

This study, therefore, examines the urban–rural gap in maternal health care services utilisation across Sub-Saharan Africa. More specifically, the study seeks to identify the factors responsible for the urban–rural difference in maternal health care services use and quantify their contribution to the gap by using an extension of the Blinder-Oaxaca decomposition technique to explain the differences in outcomes between the urban and rural population groups. Knowledge of the identified factors can help policymakers bridge the urban–rural gap in utilising maternal health care services.

## Methods

### Data source

**Fig. 1:**
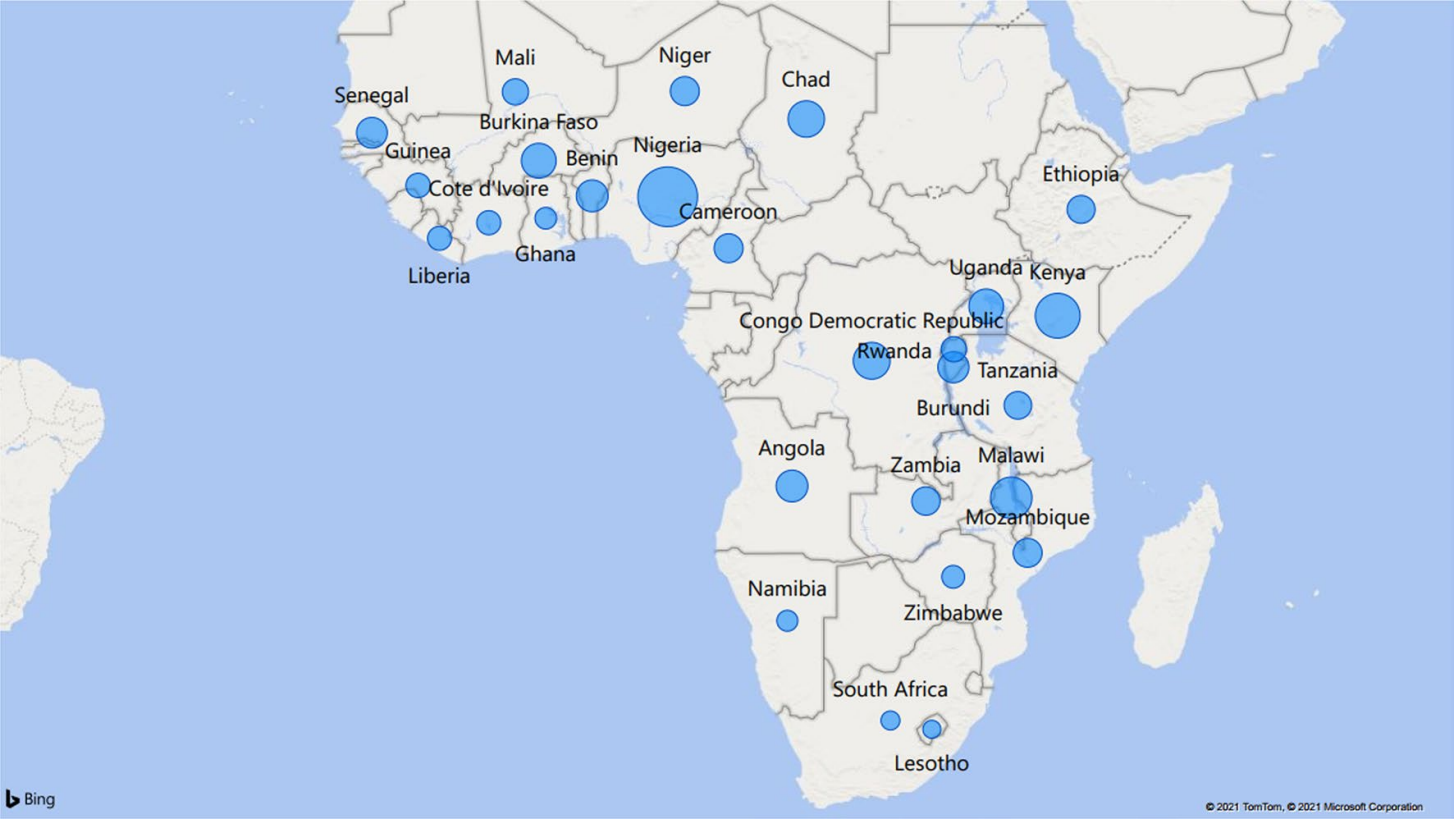
The map of 27 Sub-Saharan countries in the study

This study’s data are data collected from Demographic and Health surveys (DHS) in 27 Sub-Saharan African countries. Figure 1 gives the geographical map of the countries in the survey showing their proximity to each other and the bubble size indicating the sample size from each of the 27 countries. The sampling design of the DHS across all 27 countries was uniform, and the sample was the national representative of the individual countries. Each DHS used a two-stage stratified cluster sampling where enumeration areas were first drawn from the latest population census mapping. Then, the household samples were taken from each enumeration area. In all 27 countries, the surveys are conducted by trained interviewers using standardised questionnaires.

### Outcome variables

This study considered antenatal care, place of delivery, and professional assistance at delivery as outcomes variables. We define antenatal care as having at least four visits to a health professional by a woman after gestation for medical assessment. Her first visit must be within the first trimester of her pregnancy. The place of delivery in this study is defined as the location where a woman gives birth. Either she gives birth at home or at a health facility. We also define professional assistance at delivery as a woman being assisted by a health professional or not at the point of her delivery. By health professional, we mean a doctor, nurse, or auxiliary nurse. Since our outcomes were extracted from women’s Information on their birth histories, we based on the most recent births within 5 years before the interview to avoid recall bias.

### Independent variables

The selection of independent variables used in the analysis related to the urban–rural differences in utilisation of maternal health care services was guided by literature [27–29] and included maternal characteristics such as; *maternal age group* (15–19, 20–24, 25–29, 30–34, 35+), *maternal age at first marriage* (never married, <18, 18–24, 25+), *number of children ever born* (1, 2, 3, 4, or 5+), *wanted pregnancy* (then, later, or no more), and *current method of family plannin*g (none, modern, traditional or others). Child characteristics also were included; these are; child’s *birth order* (first, second, third, fourth, fifth, or higher) and *length of preceding birth* (first birth, < 24 months, 24–59 months, or 60+ months). Included as independent variable also are socio-economic characteristics such as; *religion* (Muslim, Christian, or Others), *woman’s current marital status* (never married, married/living together or formerly in union), *marriage or cohabitation duration* (never married, 0 to 4, 5 to 9, 10 to 14, or 15+ years), *sex of household head* (male or female), *age of husband/partner* (15–24, 25–29, 30–34, or 35+ years), *partner’s educational level* (no education, primary, secondary, or higher), *number of other wives* (never married, no other wives, or husband has other wives), *type of toilet facility* (no facility, flush toilet, pit toilet latrine or others), *The primary source of drinking water* (piped water, well water, surface water, or other sources), *woman’s Highest educational level* (no education, primary, secondary, or higher), *currently working* (no or yes), *household wealth index* (poor, middle, or rich), *media exposure* (unexposed or exposed), *final say on woman’s health care* (woman alone, woman and husband/partner, husband/partner, or someone else). As a geographical indicator, the country from which a woman comes was included as an independent variable. The independent variables used in the analysis are essential in predicting the outcomes and indicating where the urban–rural differences are in maternal healthcare services use.

### Statistical analysis

We begin our analysis by giving descriptive statistics to analyse the proportions of maternal healthcare utilisation (Antenatal care, place of delivery, and professional assistance at delivery) and urban–rural settlement across the independent variables. urban–rural differences in the independent variables were examined using the Chi-square test.

The Blinder Oaxaca decomposition has been widely utilized in economics and health in recent decades to discover and measure disparities between two groups (Blinder 1973; Oaxaca 1973). Ordinary least squares are used in this approach to decompose the difference in mean outcomes between two groups by using their additive separability. This method, however, is inapplicable when the outcome variable is binary, as with our model in this paper. As a result, we adopted a binary-model-appropriate version of the Blinder-Oaxaca approach (Fairlie [30]) to decompose the rural-urban disparity in the utilization of maternal health care services [30, 31]. The Fairlie technique entails decomposing the disparity between the mean of predicted probability instead of the mean of outcomes as in the Blinder-Oaxaca approach.

The Fairlie decomposition uses the Logit regression estimates and given below is the mathematical expression for the Fairlie decomposition.

$${\stackrel{-}{Y}}^{U}-{\stackrel{-}{Y}}^{R}=\left[\sum _{i=1}^{{N}^{U}}\frac{F\left({X}_{i}^{U}{\widehat{\beta }}^{U}\right)}{{N}^{U}}-\sum _{i=1}^{{N}^{R}}\frac{F\left({X}_{i}^{R}{\widehat{\beta }}^{U}\right)}{{N}^{R}}\right]+\left[\sum _{i=1}^{{N}^{R}}\frac{F\left({X}_{i}^{R}{\widehat{\beta }}^{U}\right)}{{N}^{R}}-\sum _{i=1}^{{N}^{R}}\frac{F\left({X}_{i}^{R}{\widehat{\beta }}^{R}\right)}{{N}^{R}}\right]$$

The first term in brackets represents the urban–rural gap in maternal healthcare utilisation due to the independent factors. The second term describes the urban–rural differences due to unmeasured factors. Details of this mathematical expression can be found in other literature [32].

This study carried out all statistical analyses using Stata version 15 (StataCorp. College Station, TX, USA). The decomposition analysis was implemented using the Fairlie package that supports non-linear decomposition for binary dependent variables [33].

## Result

### Descriptive results

Table 1 provides summary statistics for the variables used in this study. From the result, 46.1% of women in a rural area had no education against 23.6% of women in an urban area. Similarly, 39.7% of the women in rural areas have husbands/partners with no education, against 19.8% in an urban area. Also, 60.1% of the women in rural areas are from households with poor wealth index, while only 16.0% of women in urban areas are from households with poor wealth indexes. Most women in rural areas (63.6%) are not exposed to media, while only 32.5% of women in urban areas are unexposed to media.

**Table 1:** The distribution of the selected characteristics by resident area

Covariates	Urban	Rural
	n (%)	n (%)
Maternal age group		
15–19	4661 (6.8)	11954 (7.9)
20–24	15725 (22.9)	34290 (22.7)
25–29	18707 (27.2)	37454 (24.7)
30–34	14419 (21.0)	29819 (19.7)
35+	15271 (22.2)	37864 (25.0)
Maternal age at first marriage		
Never married	7431 (10.8)	8957 (5.9)
< 18	23875 (34.7)	76775 (50.7)
18–24	29719 (43.2)	57466 (38.0)
25+	7758 (11.3)	8183 (5.4)
Number children ever born		
1	17514 (25.5)	27703 (18.3)
2	15300 (22.2)	25648 (16.9)
3	11728 (17.1)	22859 (15.1)
4	8533 (12.4)	19793 (13.1)
5+	15708 (22.8)	55378 (36.6)
Wanted pregnancy		
Then	48775 (70.9)	112598 (74.4)
Later	15581 (22.7)	29030 (19.2)
No more	4427 (6.4)	9753 (6.4)
Current method of family planning		
None	43220 (62.8)	111051 (73.4)
Modern	22458 (32.7)	36175 (23.9)
Traditional	2802 (4.1)	3634 (2.4)
Others	303 (0.4)	521 (0.3)
Child's birth order		
First	17514 (25.5)	27703 (18.3)
Second	15300 (22.2)	25648 (16.9)
Third	11728 (17.1)	22859 (15.1)
Fourth	8533 (12.4)	19793 (13.1)
Fifth	5724 (8.3)	16349 (10.8)
Higher	9984 (14.5)	39029 (25.8)
Length of preceding birth		
First birth	17514 (25.5)	27703 (18.3)
< 24 months	8110 (11.8)	20962 (13.8)
24–59 months	31974 (46.5)	85706 (56.6)
60+ months	11185 (16.3)	17010 (11.2)
Religion		
Muslim	19746 (28.7)	44907 (29.7)
Christian	41638 (60.5)	87071 (57.5)
Others	7399 (10.8)	19403 (12.8)
Woman's current marital status		
Never married	7431 (10.8)	8957 (5.9)
Married or living together	55592 (80.8)	131824 (87.1)
Formerly in union	5760 (8.4)	10600 (7.0)
Marital or cohabitation duration		
Never married	7431 (10.8)	8957 (5.9)
0 to 4	16705 (24.3)	29873 (19.7)
5 to 9	17670 (25.7)	36776 (24.3)
10 to 14	12422 (18.1)	30992 (20.5)
15+	14555 (21.2)	44783 (29.6)
Sex of household head		
Male	51774 (75.3)	122164 (80.7)
Female	17009 (24.7)	29217 (19.3)
Age of husband/partner		
15–24	4171 (6.1)	11196 (7.4)
25–29	12455 (18.1)	26482 (17.5)
30–34	15268 (22.2)	30192 (19.9)
35+	36889 (53.6)	83511 (55.2)
Partner's educational level		
No education	13642 (19.8)	60124 (39.7)
Primary	16230 (23.6)	53262 (35.2)
Secondary	29600 (43.0)	33650 (22.2)
Higher	9311 (13.5)	4345 (2.9)
Number of other wives		
Never married	7431 (10.8)	8957 (5.9)
No other wives	52148 (75.8)	108004 (71.3)
Husband has other wives	9204 (13.4)	34420 (22.7)
Type of toilet facility		
No facility	7911 (11.5)	54979 (36.3)
Flush toilet	20416 (29.7)	5158 (3.4)
Pit toilet latrine	39217 (57.0)	90053 (59.5)
Others	1239 (1.8)	1191 (0.8)
The primary source of drinking water		
Piped water	39452 (57.4)	25779 (17.0)
Well water	18912 (27.5)	80826 (53.4)
Surface water	5374 (7.8)	42057 (27.8)
Other sources	5045 (7.3)	2719 (1.8)
Woman's Highest educational level		
No education	16246 (23.6)	69728 (46.1)
Primary	20015 (29.1)	56007 (37.0)
Secondary	27056 (39.3)	23786 (15.7)
Higher	5466 (7.9)	1860 (1.2)
Currently working		
No	26500 (38.5)	54654 (36.1)
Yes	42283 (61.5)	96727 (63.9)
Household wealth index		
Poor	10988 (16.0)	90955 (60.1)
Middle	8052 (11.7)	27185 (18.0)
Rich	49743 (72.3)	33241 (22.0)
Media Exposure		
Unexposed	22341 (32.5)	96216 (63.6)
Exposed	46442 (67.5)	55165 (36.4)
Final say on woman's health care		
Woman alone	10260 (14.9)	18620 (12.3)
Woman and husband/partner	33006 (48.0)	64485 (42.6)
Husband/partner	25188 (36.6)	67246 (44.4)
Someone else	329 (0.5)	1030 (0.7)

Table 2 demonstrates the discrepancies between urban and rural women in utilising maternal health care services across all covariates. In urban areas, antenatal care utilisation was greater than in rural were about 34.7 per cent of women from urban areas received antenatal care against 22.4 per cent of women in rural areas. Similarly, about 83.2 per cent of women in urban settlement gave birth to their last child at a health facility, while for women in a rural settlement, it was 58.2 per cent. Regarding professional assistance at delivery, women in rural areas were likewise disadvantaged, having 68.9 per cent of women delivering their last child with professional assistance. In comparison, for women in urban areas, 89.8 per cent of them gave birth to their last child with a health professional’s assistance.

**Table 2:** Descriptive characteristics of women utilizing maternal healthcare by urban–rural status

	Antenatal		Delivery place		Health professional gave delivery care	
	Urban	Rural	Urban	Rural	Urban	Rural
Maternal age group	(155.74)	(43.26)	(88.01)	(449.85)	(75.17)	(315.38)
15–19	5.4	7.3	6.6	8.2	6.7	8.1
20–24	22.3	23.1	22.9	24.2	23.0	23.6
25–29	28.5	25.4	27.6	24.5	27.5	24.7
30–34	22.0	20.0	21.2	19.7	21.1	19.6
35+	21.8	24.2	21.7	23.5	21.8	23.9
Maternal age at first marriage	(761.79)	(885.80)	(1694.82)	(5324.80)	(1063.46)	(3579.47)
Never married	10.7	7.1	11.3	7.6	11.1	6.9
< 18	29.3	43.9	31.4	43.0	32.8	45.6
18–24	45.0	42.0	44.9	42.8	44.3	41.4
25+	14.9	7.0	12.3	6.5	11.9	6.1
Number children ever born	(665.69)	(636.00)	(1900.39)	(1268.34)	(2713.18)	
1	29.2	21.1	27.5	22.5	26.7	20.9
2	24.0	18.6	23.1	18.5	22.8	17.9
3	17.4	16.1	17.3	15.5	17.2	15.5
4	11.4	13.0	12.1	12.7	12.2	12.8
5+	17.9	31.2	20.0	30.9	21.1	33.0
Wanted pregnancy	(156.11)	(16.20)	(112.70)	(177.58)	(1923.60)	
Then	73.8	73.8	70.1	69.8	70.1	71.1
Later	20.8	19.9	23.4	22.9	23.3	21.8
No more	5.4	6.3	6.5	7.3	6.5	7.1
Current method of family planning	(260.44)	(1404.73)	(1891.19)	(7897.62)	(1452.98)	(5774.34)
None	58.8	65.6	59.2	64.8	60.5	67.6
Modern	35.8	31.4	35.8	31.7	34.7	29.2
Traditional	4.9	2.5	4.5	3.1	4.3	2.8
Others	0.5	0.4	0.5	0.4	0.5	0.4
Child's birth order	(698.52)	(689.13)	(2062.46)	(4447.23)	(1366.42)	(2865.13)
First	29.2	21.1	27.5	22.5	26.7	20.9
Second	24.0	18.6	23.1	18.5	22.8	17.9
Third	17.4	16.1	17.3	15.5	17.2	15.5
Fourth	11.4	13.0	12.1	12.7	12.2	12.8
Fifth	7.2	10.2	7.8	10.0	8.0	10.3
Higher	10.7	21.0	12.2	20.9	13.1	22.6
Length of preceding birth	(518.19)	(758.19)	(1518.38)	(4326.72)	(964.93)	(2671.16)
First birth	29.2	21.1	27.5	22.5	26.7	20.9
< 24 months	9.5	11.0	10.7	11.2	11.2	12.4
24–59 months	43.2	54.1	44.4	53.1	45.2	54.1
60+ months	18.1	13.8	17.4	13.3	17.0	12.6
Religion	(43.58)	(1212.86)	(1248.58)	(9316.70)	(1190.36)	(7567.68)
Muslim	27.8	24.3	26.0	21.6	26.7	24.4
Christian	62.2	65.7	62.8	67.8	62.2	64.9
Others	10.0	10.0	11.2	10.6	11.1	10.6
Woman's current marital status	(12.68)	(113.78)	(111.24)	(1367.92)	(45.45)	(703.64)
Never married	10.7	7.1	11.3	7.6	11.1	6.9
Married or living together	81.4	85.7	80.5	84.6	80.7	85.7
Formerly in union	7.9	7.2	8.2	7.7	8.3	7.4
Marital or cohabitation duration	(260.28)	(363.89)	(778.98)	(3228.56)	(549.64)	(2096.15)
Never married	10.7	7.1	11.3	7.6	11.1	6.9
0 to 4	26.9	22.0	25.6	22.9	25.1	21.8
5 to 9	26.6	24.6	25.8	24.4	25.8	24.5
10 to 14	17.4	19.6	17.7	19.0	17.9	19.6
15+	18.4	26.7	19.5	26.0	20.1	27.1
Sex of household head	(8.63)	(83.99)	(30.78)	(816.25)	(4.09)	(328.84)
Male	74.6	79.0	74.9	78.2	75.2	79.5
Female	25.4	21.0	25.1	21.8	24.8	20.5
Age of husband/partner	(126.10)	(102.8)	(165.41)	(2234.46)	(187.22)	(1771.45)
15–24	4.9	7.6	6.0	8.9	6.1	8.4
25–29	17.7	18.5	18.5	19.7	18.5	19.1
30–34	23.7	21.1	22.9	21.0	22.7	20.7
35+	53.8	52.8	52.6	50.4	52.8	51.8
Partner's educational level	(1126.33)	(1604.19)	(2976.98)	(11,536.30)	(2303.29)	(8857.36)
No education	16.5	32.0	16.6	28.5	17.6	31.9
Primary	20.1	35.9	22.8	39.8	23.2	38.5
Secondary	44.8	27.9	45.4	27.8	44.8	26.1
Higher	18.6	4.3	15.1	3.8	14.4	3.5
Number of other wives	(111.38)	(404.55)	(840.49)	(3491.84)	(488.97)	(2670.86)
Never married	10.7	7.1	11.3	7.6	11.1	6.9
No other wives	77.7	73.9	76.9	74.4	76.5	73.9
Husband has other wives	11.5	19.0	11.7	17.9	12.4	19.2
Type of toilet facility	(1173.38)	(764.54)	(1849.67)	(9169.66)	(1190.89)	(3463.61)
No facility	9.4	31.9	9.4	26.4	10.4	31.5
Flush toilet	37.8	5.3	31.9	4.4	31.3	4.0
Pit toilet latrine	51.1	61.8	56.9	68.4	56.5	63.6
Others	1.6	0.9	1.8	0.8	1.8	0.8
The primary source of drinking water	(653.98)	(550.26)	(2909.73)	(2331.64)	(2133.14)	(1512.59)
Piped water	62.2	21.0	61.9	20.9	60.3	19.5
Well water	23.9	50.8	24.5	50.2	25.7	51.4
Surface water	5.4	26.0	6.7	26.9	7.1	27.3
Other sources	8.5	2.1	6.9	2.0	6.9	1.9
Woman's Highest educational level	(1354.01)	(2050.50)	(4380.38)	(15,386.76)	(3522.65)	(12,317.41)
No education	19.2	37.0	19.4	33.2	20.6	36.8
Primary	25.3	39.4	28.4	43.3	29.1	42.0
Secondary	43.5	21.5	42.9	21.6	41.6	19.6
Higher	11.9	2.1	9.2	1.9	8.7	1.7
Currently working	(49.09)	(177.76)	(70.63)	(1704.62)	(10.50)	(723.42)
No	36.7	33.0	37.8	31.8	38.3	33.9
Yes	63.3	67.0	62.2	68.2	61.7	66.1
Household wealth index	(745.27)	(612.77)	(5318.88)	(5788.88)	(5889.99)	(8922.42)
Poor	11.5	56.0	12.1	52.4	13.1	52.7
Middle	10.0	17.2	10.1	19.6	10.2	19.3
Rich	78.5	26.8	77.8	27.9	76.7	28.0
Media Exposure	(837.52)	(1202.37)	(2027.92)	(4245.84)	(1450.63)	(2248.54)
Unexposed	25.4	55.6	28.9	56.7	30.2	59.6
Exposed	74.6	44.4	71.1	43.3	69.8	40.4
Final say on woman's health care	(85.96)	(710.59)	(636.74)	(4133.76)	(734.67)	(4534.15)
Woman alone	15.5	13.9	15.4	13.8	15.4	13.7
Woman and husband/partner	49.8	47.3	49.5	48.0	49.2	47.0
Husband/partner	34.3	38.1	34.5	37.5	34.9	38.7
Someone else	0.4	0.7	0.5	0.7	0.5	0.6

Figures in parentheses are Chi-square values; all values were significant at p < 0.1 level of significance

Furthermore, Table 2 indicates that all the assessed characteristics are related to maternal health services in urban and rural areas. Maternal age was associated with antenatal care, delivery place, and professional assistance at delivery. The prevalence of these health care services’ use was more among older women than the younger women in urban and rural areas.

The woman’s educational level was associated with the use of health care services. About 55 per cent of urban women having secondary or higher education attended at least four antenatal care visits, with the first visit within the first trimester. Only about 24 per cent of women in rural areas with such education levels attended at least four antenatal visits. About 52 per cent of the women of urban regions that attained a secondary or higher educational level delivered their last child at a health facility. In comparison, only about 24 per cent of women had the same educational level and gave birth to their last child at a health facility. Similarly, about 50 per cent of the women from urban settlements who gave birth with health professionals’ assistance had secondary or higher education. In comparison, only about 21 per cent of women from the rural area that a health professional assisted at delivery have secondary or higher education.

Women that are working utilise health care services more than women that are not working; this was true for both urban and rural settlements, as seen in Table 3, where the percentage of those that use health care services were all higher among those working. The use of health care services was more among women who joined with their husband or partner to have the final say on their health care across all outcomes, both for rural and urban cases than for any other group.

### Decomposition result

Table 3 presents a summary of the decomposition analysis for the rural-urban gap in the use of maternal healthcare services (antenatal care, delivery place, and professional assistance at delivery). The rural-urban disparity in health care services use was 12.2 per cent for antenatal care, 25 per cent for the delivery place, and 20.9 per cent for professional assistance at delivery. The differences in the covariates included in our analysis explained about 75 per cent of the urban–rural disparity in antenatal care utilisation. Similarly, the covariates’ difference explained about 79 per cent of the urban–rural gap in the use of health facility for delivery. Likewise, these covariates explained about 82 per cent of the disparity in professional assistance at delivery utilisation.

**Table 3:** Decomposition of the measured maternal healthcare services

	Antenatal care	Place of delivery	Professional assistance at delivery
Proportion Urban	0.347	0.832	0.898
Proportion Rural	0.224	0.582	0.689
Differences in proportion (Urban–Rural)	0.122	0.250	0.209
Total proportion explained	0.093	0.194	0.172
Percentage explained	76.3	77.6	82.4

Table 4 shows the detailed decomposition of individual characteristics’ contribution to urban–rural inequality in maternal health care utilisation. A negative contribution will suggest that the specific variable eliminates the urban–rural difference in maternal healthcare services use and vice versa. Household wealth index, mother’s education, husband/partners education, media exposure, and childbirth order were among the top contributors to the average urban–rural disparity in the use of all the three healthcare services. For antenatal care, the household wealth index contributed to 20.7 per cent of the urban–rural difference, 18.5 per cent by exposure to media, 16.3 per cent by childbirth order, and 15.2 per cent by maternal and husband/partner education.

**Table 4:** Effect and contribution of each predictor variable in the urban–rural difference in utilisation of maternal healthcare service in Sub-Saharan Africa

Covariates	Antenatal care		Place of delivery		Professional assistance at delivery	
	Coefficients	%Contribution	Coefficients	%Contribution	Coefficients	%Contribution
Mothers age	−0.002***	−1.7	0.001***	0.5	0.001***	0.4
Age at first marriage	0.002***	1.8	0.006***	3.2	0.004***	2.5
Number of children	0.004*	4.4	0.001**	0.8	0.001*	0.6
Wanted child	−0.001***	−0.6	0.002***	1	0.002***	1.1
Family planning method	0.004***	4.3	0.009***	4.8	0.007***	4.2
Childbirth order	0.015***	16	0.014***	7.1	0.007***	4
Birth interval	< 0.001***	−0.5	−0.001***	−0.4	< 0.001***	−0.2
Religion	< 0.001***	0.3	0.001***	0.6	0.002***	1.1
Marital Status	0.001***	0.6	< 0.001***	0.2	< 0.001**	0
Duration of marriage	−0.001	−0.8	−0.001**	−0.7	−0.001***	−0.6
Sex of head of household	0.001***	0.6	0.002***	0.8	< 0.001*	0.1
Husband's age	< 0.001***	0.3	0.001***	0.3	0.001***	0.5
Husband's educational level	0.013***	14.3	0.013***	6.9	0.005***	3
Husband's wives	0.002***	2.3	0.005***	2.7	0.003***	1.9
Toilet type	−0.002***	−1.8	0.009***	4.8	−0.001**	−0.4
Source of drinking water	0.004***	4.6	0.008***	4.1	0.005***	3.1
Mothers educational level	0.014***	15.1	0.037***	19.1	0.029***	17.1
Work status	< 0.001***	−0.4	−0.002***	−1.1	< 0.001***	−0.2
Wealth index	0.019***	20.2	0.072***	37	0.092***	53.3
Exposure to media	0.018***	19.2	0.019***	9.6	0.01***	5.7
Decision on woman’s health	0.001***	1.2	0.002***	1.1	0.004***	2.1
Country	0.001***	0.7	−0.005***	−2.5	0.001***	0.6

Level of significance: *p < 0.10, **p < 0.05, ***p < 0.01

For the outcome place of delivery, household wealth index contributed to 34.3 per cent of the urban–rural difference, 19.2 per cent by mother’s education, 10.6 per cent by media exposure, 7.1 per cent by childbirth order, and 5.6 per cent by husband/partners education. For professional assistance at delivery, household wealth index contributed to 54.4 per cent of the urban–rural difference, 17 per cent by maternal education, media exposure contributed to 5.3 per cent, childbirth order contributed to 4.1, and husband/partners education contributed to 3.5 per cent of the difference.

## Discussion

This study measured the inequality in maternal health care services utilisation using pooled data from 27 Sub-Saharan Africa countries. The urban–rural difference in maternal healthcare service and its contributing factors was determined using a non-linear extension of the Blinder-Oaxaca decomposition technique. We found that there is a significant difference in maternal health care use between urban and rural areas. The level of maternal healthcare services utilisation in the rural area was far lower than that of the urban area. This finding is consistent with most past studies in this area [14, 34–37].

Health care facilities in rural regions of Sub-Saharan Africa are typically in a poor state, which may contribute to the low utilization of maternal healthcare services [38]. It is challenging to find competent healthcare personnel in rural regions, even when the facilities are accessible. Low motivation discourages qualified healthcare workers from working in remote areas [39, 40]. Health care facilities in rural areas are usually located centrally, making it difficult for many women in such areas to access them easily, thereby causing a low use of such services.

In the decomposition analysis, household wealth index, exposure to media, educational level for mothers and their partner, and childbirth order contributed the most towards explaining the urban–rural disparity in maternal health care use. Except for childbirth order, all of the other top contributing factors are socio-economic variables, which shows that rural areas’ socio-economic conditions help widen the urban–rural gap in maternal healthcare utilisation. Similar findings have been reported in other developing countries [41].

According to our findings, the most significant urban–rural divide exists in the usage of place of delivery. Women from rural regions prefer to give birth at home or other centers rather than a health institution. This discovery aligns with earlier studies [42, 43]. In some cases, these health facilities are not within a distance that can easily be accessible by women in the rural areas, and the cost of transportation, which most of the women cannot afford, makes them prefer to give birth at home. In most cases, delivery at health care facilities in rural areas is free. Still, there is not enough sensitisation on that. Governments make most of their effort on media where women in rural areas are not mostly exposed to or are not educated enough to understand the message.

Professional health workers such as doctors, nurses, and auxiliary nurses are in high demand, even in urban areas [44]. Therefore, it will take much motivation and incentives to make these health professionals available in rural areas; this shed some light on the urban–rural difference in the use of professional assistance at delivery. The poor socio-economic condition of women in rural areas means most of them cannot afford to go to where they can be assisted by health professionals when giving birth. Indeed, they cannot get them available when delivering at home as most healthcare professionals are situated in urban areas.

The WHO guideline for antenatal care was utilized in this study. After gestation, it is expected that a woman will have at least four antenatal care appointments, with the first visit occurring during the first trimester of her pregnancy [45]. This WHO recommendation is hardly met even by women in a rural area in Sub-Saharan Africa, explaining why the urban–rural difference was the least among the three measured maternal health care services. Even when these antenatal care services are free for women in rural areas, the cost of transportation to these services means that many women in rural areas cannot afford to meet the recommendation [46]. The lack of exposure to media and low educational level, even of their husbands or partners, means they cannot afford the necessary Information to make them see the need for such services.

## Strengths and limitations

This study’s major strength is that it uses nationally representative data from various Sub-Saharan Africa countries, making the region’s findings generalisable. Another strength of the study is the non-linear decomposition technique used, making it possible to quantify the effect of the factors identified in the urban–rural gap, unlike other previous studies that only identify the factors responsible for the disparity.

However, a significant drawback is that the study’s outcomes are based on a mother’s subjective report. In as much as we used the most recent births, we cannot completely rule out bias in reporting by the mothers. Another drawback of the study is the inability to establish a causal relationship, given that the study used cross-sectional data for analysis and the independent factors are related with the outcome of interest.

## Conclusions

This study analysed the urban–rural differences in maternal health care services utilisation using nationally representative Demographic Health Survey data from 27 Sub-Saharan countries. The Fairlie decomposition technique, an extension of the popular Blinder-Oaxaca decomposition method, helped us identify the factors that cause this urban–rural gap and quantify their impact in widening the urban–rural gap in maternal healthcare utilisation.

The study found a substantial disparity between rural and urban areas in the use of antenatal care, health facility for delivery, and health professional assistance at delivery to the rural areas’ disadvantage. Socio-economic factors mostly explain the disparity; mothers from a household with a rich wealth index are more likely to be found in urban areas and are more likely to use maternal healthcare services. Higher educational and media exposure are characteristics of urban women, making them more likely to use maternal healthcare services than rural women.

The continuation of substantial rural-urban gaps in maternal healthcare services utilisation reflects the inability of social and health strategies to achieve sustained improvement in health for all demographic groups. Overall, to reduce the urban–rural disparity in the use of maternal health services in Sub-Saharan Africa, policy interventions must take into account factors such as economic status, level of education, and media exposure.

## Data Availability

The dataset supporting the conclusions of this article is available in the IDHS repository. https://www.idhsdata.org/idhs-action/menu.

## Abbreviations

DHS: Demographic Health Survey
WHO: World Health Organisation
